# The Spatial Organization of DNA Virus Genomes in the Nucleus

**DOI:** 10.1371/journal.ppat.1003386

**Published:** 2013-06-27

**Authors:** Roger D. Everett

**Affiliations:** MRC-University of Glasgow Centre for Virus Research, Glasgow, United Kingdom; University of Florida, United States of America

## Where Do the Genomes of DNA Viruses Go When They Enter the Nucleus?

During lytic infection, DNA viruses that replicate in the nucleus of cells establish an environment that favors viral replication while evading cellular defenses. Consequently, these viruses must engage with cellular factors that are necessary for efficient viral transcription and DNA replication, while disarming restrictive cellular responses to the presence of the invading pathogenic DNA. It is important to consider these events not only at the biochemical level of protein-protein and protein-nucleic acid interactions, but also in terms of spatial organization within the nucleus. The nucleus contains many distinct substructures, including nucleoli, chromosome territories, splicing speckles, PML Nuclear Bodies (PML NBs, also known as ND10), and transcription sites, all of which are in a dynamic environment involving the exchange of protein molecules between the structures themselves and the general nucleoplasm [1]. DNA viruses establish their own transcription sites and replication compartments within the nucleus, and a topic that has been of interest for many years is how these virus-specific loci relate to the preexisting cellular nuclear substructures, particularly during the early stages of infection. Is viral genome localization random, or is it regulated in some way?

Gerd Maul made a breakthrough discovery when he found that after their delivery into the nucleus, the genomes of many different DNA viruses are frequently associated with PML NBs [2], [3]. These intriguing findings posed many questions: How does this association occur? What are the viral and cellular signals involved? Does the association have a positive or negative influence on infection?

## Viral Genome Association with PML NBs—A Cellular Response?

**Figure 1:**
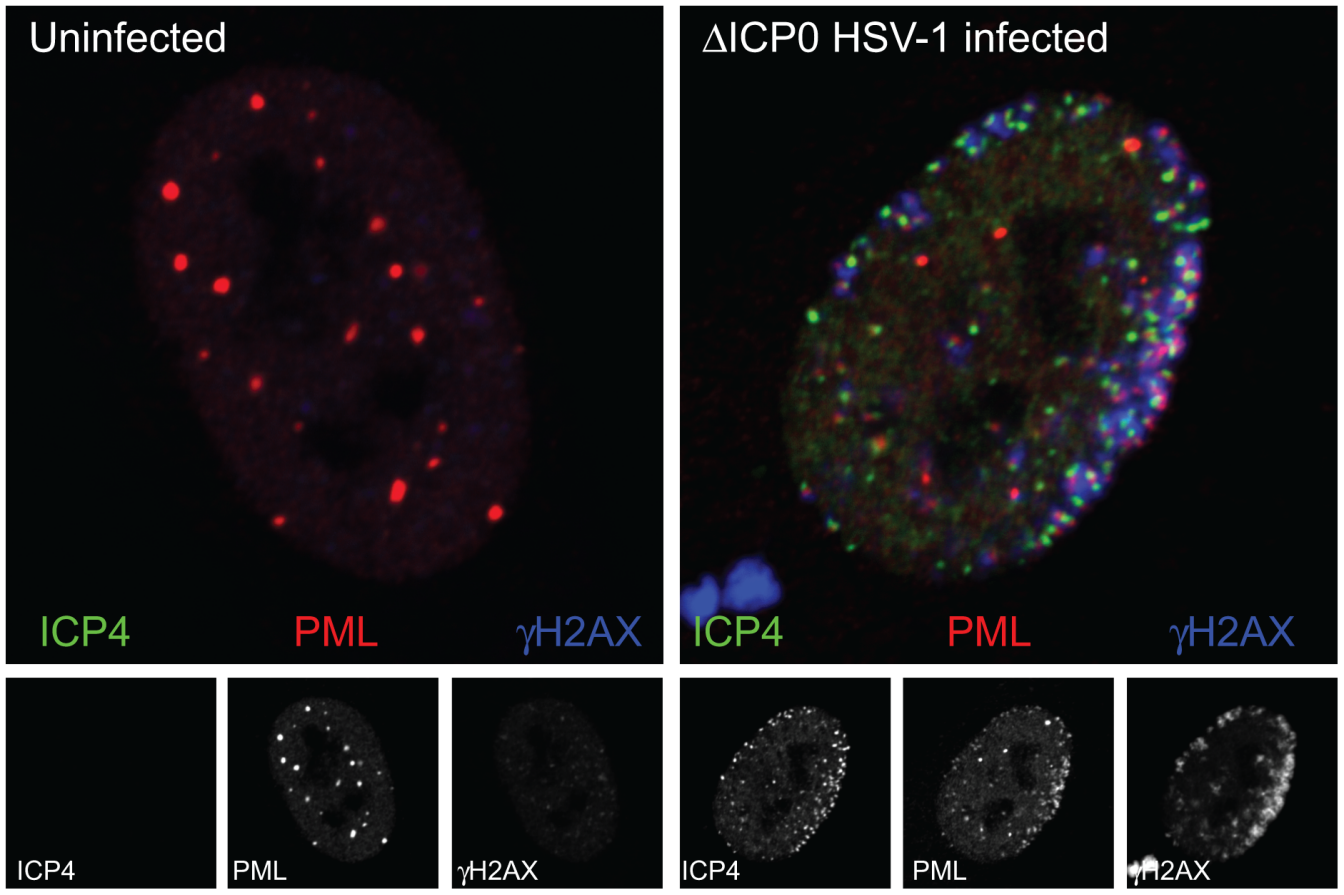
The association of PML and DNA damage response foci with HSV-1 genomes. The uninfected human diploid fibroblast nucleus on the left shows a typical distribution of PML NBs (PML, red) with only faint staining of the DNA damage response protein γH2AX (blue), with the separated channels shown in grayscale below. The infected cell nucleus on the right is of a cell at the edge of a developing ICP0-null mutant HSV-1 plaque, which has been infected with a very high number of virus particles by spread from neighboring heavily infected cells. The viral genomes can be detected by the presence of the viral transcriptional activator ICP4 (green), which binds efficiently to viral DNA. PML (red) has been redistributed from its normal locations to sites that closely associated with the viral genomes. This association is stabilized by the absence of ICP0, which efficiently inhibits the formation of these foci in a normal wild type HSV-1 infection. A DNA damage response has been initiated to produce a marked increase in γH2AX (blue) in regions close to the viral genomes.

Initially it was thought that viral genomes might migrate through the nucleoplasm until they engaged with preexisting PML NBs. While this has not been excluded, particularly for the smaller DNA viruses, this model presents some practical problems for large DNA virus genomes. For example, the genomes of herpesviruses, in excess of 150 kbp, are likely to have limited mobility in the nucleoplasm, especially as they will rapidly accumulate chromatin-related and other binding partners and thus achieve significant bulk and mass. Indeed, there is strong evidence that HSV-1 genomes do not move far from the inner nuclear membrane after their entry into the nucleus through the nuclear pore [4], [5]. PML NBs are also of substantial size (of the order of 1 µm diameter), but while the structures themselves have limited mobility, the component proteins undergo rapid exchange with the general nucleoplasm [6]. Therefore an alternative explanation of viral genome–PML NB association is that it occurs not because either entity migrates through the nucleoplasm, but because new PML NBs are formed at the sites of the viral genomes due to the deposition of PML NB protein molecules. In the case of HSV-1, a chance observation provided very strong evidence that this was indeed the case—the viral genomes became very rapidly associated with novel PML NB–like structures in the earliest stages of infection through recruitment of PML NB proteins [4] (Figure 1). While for technical reasons it is more difficult to confirm that this is also the case in other DNA virus infections, it is likely that analogous events occur more generally. If so, the association of viral genomes and PML NB–like structures can be viewed as a cellular response to the entry of a viral genome into the nucleus.

## What Factors Are Involved in the Association between DNA Virus Genomes and PML NBs?

The issues that influence the association between DNA virus genomes and PML NBs include the virus factors that are required for the response, the properties of PML NB proteins required for their recruitment, and the actions of viral regulatory proteins that affect the stability of the recruitment. Because the association of adenovirus and HSV-1 genomes with PML NBs occurs when viral transcription and protein expression are inhibited [2]–[4], it appears that the viral genome itself may be sufficient to trigger the response. It is possible that the initial signal involves unchromatinized “foreign” DNA, but note that PML NB components can also be recruited to unusual chromosomal locations such as integrated transgene arrays with multiple regulatory factor binding sites [7].

From the cellular aspect, although PML is absolutely required for the assembly of PML NBs in uninfected cells [8], a number of other PML NB proteins (such as Sp100, hDaxx, ATRX, and SUMO-2/3) become associated with HSV-1 genomes even in cells highly depleted of PML [9]–[12]. There is evidence that similar events occur in HCMV-infected cells [13]. The assembly and dynamics of PML NBs are strongly linked to modification by the ubiquitin-like SUMO family of proteins, and this also holds true for the viral genome–associated structures. Recruitment of PML requires both its sumoylation and its ability to interact noncovalently with SUMO through its SUMO interaction motif (SIM) [12], and recruitment of both Sp100 and hDaxx requires their SIMs [12]. ATRX on the other hand is recruited through its interaction with hDaxx [11]. Therefore the association requires viral genomes on the one hand, and a cascade of events involving both sumoylation-dependent and -independent events on responsive cellular proteins on the other.

An important factor involved in the association of DNA viral genomes and PML NBs is the effect of viral regulatory proteins on their stability. The recruitment is very short-lived in normal wild type HSV-1 infection because the viral immediate-early protein ICP0, an E3 ubiquitin ligase, induces degradation of PML and disruption of PML NBs [14], [15]. However, in the absence of ICP0, the recruitment is stable and easily visualized [4] (Figure 1). The association between PML NBs and the genomes of HCMV and adenovirus is also most readily observed at early times of infection or with mutant viruses that fail to express regulatory proteins that would normally disrupt PML NBs [2], [16]. In contrast, in cultured cells harboring quiescent HSV-1 [9] and in latently infected mouse neurons [17] (conditions in which ICP0 is not expressed), HSV-1 genomes are surrounded by a shell of PML NB proteins. It is possible that this reflects sequestration of the viral genomes in an inactive, repressed environment.

## Is the Recruitment of PML NB Proteins to Viral Genomes Linked to a DNA Damage Response?

The initial stages of the recruitment process are of obvious interest. Because DNA viral genomes in the initial stages of infection are in a state quite distinct from cellular chromatin, it is possible that a DNA damage response (DDR) is involved. Many DNA viruses induce a DDR [18], which in some cases involves the accumulation of DNA repair proteins in close proximity to viral DNA [19], [20], or within developing viral replication compartments (reviewed in [18]) (see also Figure 1). Furthermore, PML has been observed to accumulate at sites of DNA damage [21]. PML NBs have been suggested to act as sensors for damaged DNA [22], they include proteins involved in both DNA repair and chromatin modification [8], and sumoylation events are involved in both the recruitment of PML NB proteins to HSV-1 genomes [12] and the DDR [23]. Despite these parallels, it remains unclear whether PML NB and DNA damage responses during the initial stages of DNA virus infection are related. Although ICP0 of HSV-1 inhibits both responses, this occurs through distinct mechanisms [19]. Furthermore, the formation of DDR foci close to HSV-1 genomes does not require PML or other major PML NB components, and is also atypical of classical DDR because it does not require Mre11 or ATM [19]. Therefore the association of DNA viral genomes with PML NBs and DDR foci may occur through distinct mechanisms. Whether other DNA sensors, such as IFI16, are involved in the PML NB response remains to be determined. It is noteworthy that IFI16 becomes associated with herpesvirus genomes during infection [24], [25].

## What Are the Consequences of Viral Genome Association with Cellular Nuclear Substructures and Protein Complexes?

Whether the association between DNA virus genomes and PML NBs has a positive or negative influence on the outcome of infection has long been debated. On the one hand, viral transcription and replication sites develop initially in association with PML NB–like structures [16], which could indicate a beneficial effect. On the other hand, the defective phenotype of virus mutants unable to disrupt PML NBs suggests a negative influence, and it is this view that dominates current thinking. RNAi-mediated depletion of PML NB proteins has revealed several examples of herpesvirus infections that are restricted by one or more PML NB components, and in these cases the viruses express regulatory proteins that overcome the restriction [26]. There is evidence in the case of HSV-1 that the DDR also contributes to initial restrictive responses to viral DNA [19], although in general DDR proteins play both positive and negative roles during DNA virus infection [18]. The degree of restriction imparted by any one PML NB protein may be modest, but there is evidence that PML NB–mediated inhibition of herpesvirus infections involves the cooperative actions of several PML NB components [27], [28].

In conclusion, the current information is consistent with the hypothesis that entry of DNA virus genomes into the nucleus stimulates a cellular response that leads to the accumulation of PML NB proteins, and probably other restrictive proteins yet to be identified, in close association with the viral genomes. In turn, viruses express regulatory proteins that inactivate this defensive response and thus stimulate lytic infection. Thus the outcome of DNA virus infection is governed not only by the biochemical properties of cellular and viral regulatory proteins, but also by the dynamic manner in which they are spatially organized in the nucleus.
